# *Sleeping Beauty* Transposon Mutagenesis as a Tool for Gene Discovery in the NOD Mouse Model of Type 1 Diabetes

**DOI:** 10.1534/g3.115.021709

**Published:** 2015-09-30

**Authors:** Colleen M. Elso, Edward P. F. Chu, May A. Alsayb, Leanne Mackin, Sean T. Ivory, Michelle P. Ashton, Stefan Bröer, Pablo A. Silveira, Thomas C. Brodnicki

**Affiliations:** * Immunology and Diabetes Unit, St. Vincent’s Institute, Fitzroy, Victoria 3065, Australia; † Department of Medicine, University of Melbourne, Parkville, Victoria 3010, Australia; ‡ Research School of Biology, The Australian National University, Canberra, ACT 2601, Australia; § Dendritic Cell Research, ANZAC Research Institute, Concord, New South Wales 2139, Australia

**Keywords:** forward genetics, reverse genetics, *Slc16a10*, *Serinc1*, amino acid transporter

## Abstract

A number of different strategies have been used to identify genes for which genetic variation contributes to type 1 diabetes (T1D) pathogenesis. Genetic studies in humans have identified >40 loci that affect the risk for developing T1D, but the underlying causative alleles are often difficult to pinpoint or have subtle biological effects. A complementary strategy to identifying “natural” alleles in the human population is to engineer “artificial” alleles within inbred mouse strains and determine their effect on T1D incidence. We describe the use of the *Sleeping Beauty* (*SB*) transposon mutagenesis system in the nonobese diabetic (NOD) mouse strain, which harbors a genetic background predisposed to developing T1D. Mutagenesis in this system is random, but a green fluorescent protein (GFP)-polyA gene trap within the *SB* transposon enables early detection of mice harboring transposon-disrupted genes. The *SB* transposon also acts as a molecular tag to, without additional breeding, efficiently identify mutated genes and prioritize mutant mice for further characterization. We show here that the *SB* transposon is functional in NOD mice and can produce a null allele in a novel candidate gene that increases diabetes incidence. We propose that *SB* transposon mutagenesis could be used as a complementary strategy to traditional methods to help identify genes that, when disrupted, affect T1D pathogenesis.

Type 1 diabetes (T1D) is an autoimmune disease in which lymphocytes mediate the specific destruction of insulin-producing pancreatic β cells (Bluestone *et al.* 2010). Genetic studies in human populations have detected >40 genomic intervals that harbor T1D-associated alleles (Polychronakos and Li 2011; Pociot *et al.* 2010). However, identification of the underlying genes for these T1D loci and the biological effects of putative causative alleles is often difficult due to genetic heterogeneity and limited tissue availability (Polychronakos and Li 2011; Pociot *et al.* 2010). Instead, it has proven useful to complement human genetic studies with strategies that not only aim to discover “naturally” occurring alleles but also engineer “artificial” null alleles in putative and novel candidate genes to determine their effect on disease pathogenesis in inbred animal models (Ermann and Glimcher 2012).

The nonobese diabetic (NOD) mouse strain, in particular, has been widely used to investigate T1D pathogenesis (Driver *et al.* 2011; Jayasimhan *et al.* 2014). NOD mice spontaneously develop T1D, and genetic studies have identified >40 murine T1D susceptibility loci [termed insulin-dependent diabetes (*Idd*) loci], several of which overlap human T1D susceptibility loci (Burren *et al.* 2011). Although congenic NOD mouse strains have confirmed the majority of these *Idd* loci, relatively few of the underlying genes and their causative alleles have been definitively identified (Araki *et al.* 2009; Hamilton-Williams *et al.* 2001; Hung *et al.* 2006; Kissler *et al.* 2006; Laloraya *et al.* 2006; McGuire *et al.* 2009; Razavi *et al.* 2006; Tan *et al.* 2010; Yamanouchi *et al.* 2007). It has become apparent, however, that the NOD mouse strain has a combination of rare alleles (*e.g.*, *H2-Ab^g7^*) and common alleles (*e.g.*, *H2-E^null^* and *B2m^a^*) for different genes that interact and increase the risk for T1D (Driver *et al.* 2011; Ridgway *et al.* 2008). Intriguingly, some nondiabetic mouse strains harbor a more diabetogenic allele than NOD mice for a given *Idd* locus (Brodnicki *et al.* 2003; Wang *et al.* 2014; Ghosh *et al.* 1993; McAleer *et al.* 1995). This complex genetic architecture for T1D susceptibility in the *Mus* species is similar to that described in humans and further complicates the identification of “natural” causative alleles within genes underlying *Idd* loci using traditional outcross and congenic mouse studies (Driver *et al.* 2011; Ridgway *et al.* 2008).

Here, we propose an alternative approach for disease gene discovery using the *Sleeping Beauty* (*SB*) transposon mutagenesis system to generate “artificial” alleles on the NOD genetic background. The *SB* transposon can insert within genes and disrupt transcript expression (Horie *et al.* 2003; Carlson *et al.* 2003; Ivics *et al.* 1997). Its unique sequence also serves as a molecular tag to rapidly identify the site of insertion without additional breeding. Transposition (*i.e.*, transposon “jumping”) is catalyzed by the *SB* transposase, which can be expressed in *trans* and controlled by tissue-specific promoters to restrict transposition and subsequent gene mutation to germline or somatic cells. Once activated, transposition is relatively random, requiring only a target TA dinucleotide integration site and exhibiting some bias toward “jumping” in *cis*, *i.e.*, within the same chromosome (Keng *et al.* 2005; Carlson *et al.* 2003; Horie *et al.* 2003). A major advantage of the *SB* transposon is its ability to carry gene-trap elements and reporter genes, which increase gene disruption efficiency and accelerate identification of mice with a disrupted gene (Izsvak and Ivics 2004; Horie *et al.* 2003). Due to these unique characteristics, this system has been successfully used to mutate and characterize both putative and novel genes in different mouse models of cancer (Moriarity and Largaespada 2015; Dupuy *et al.* 2009). We show here that a relatively small-scale mutagenesis screen using the *SB* transposon, combined with disease-specific prioritization criteria, is able to identify a novel candidate gene that contributes to T1D susceptibility in NOD mice.

## Materials and Methods

### Constructs and production of transgenic and *SB* transposon mutant mice

**Figure 1:**
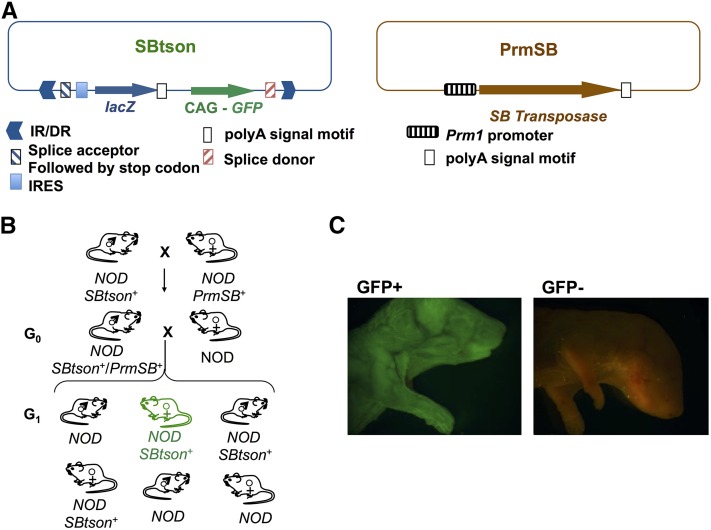
*Sleeping Beauty* transposon mutagenesis strategy. (A) Constructs used for production of NOD-*PrmSB* and NOD-*SBtson* lines. The transposon construct, pTrans-SA-IRESLacZ-CAG-GFP_SD:Neo, has been described (Horie *et al.* 2003). The transposase construct pRP1345 comprising *SB10* transposase driven by the mouse proximal protamine 1 promoter has also been described (Fischer *et al.* 2001). IR/DR: inverse repeat/direct repeat transposase recognition motifs. (B) Breeding scheme for *SB* transposon mutagenesis. NOD-*SBtson* mice (lines 1 and 2) were mated to NOD-*PrmSB* mice (lines 1 and 2). Double positive male offspring (seed mice) were backcrossed to wild-type NOD females to produce G_1_ mice carrying potential transposon insertions. (C) Mice carrying transposon insertions that activated the polyA trap were detected by fluorescence under UV light prior to weaning.

The transposase construct pRP1345 (Figure 1A) was obtained from Prof. R. Plasterk (Hubrecht Laboratory, The Netherlands) and has been previously described (Fischer *et al.* 2001). The transposon construct, pTrans-SA-IRESLacZ-CAG-GFP_SD:Neo (Figure 1A), was obtained from Prof. J. Takeda (Osaka University, Japan) and has been previously described (Horie *et al.* 2003). *SB* transposon and *SB* transposase transgenic mice were produced by pronuclear injection of linearized constructs into fertilized NOD/Lt (NOD) oocytes at The Walter and Eliza Hall Institute Central Microinjection Service using a previously described protocol (Marshall *et al.* 2004). Transposon transgenic mouse lines are NOD-*TgTn*(*sb-Trans-SA-IRESLacZ-CAG-GFP-SD:Neo)1Tcb* and NOD-*TgTn*(*sb-Trans-SA-IRESLacZ-CAG-GFP-SD:Neo)2Tcb*, but have been termed NOD-*SBtson* L1 and L2 in the text. Transposase transgenic mouse lines are NOD-*Tg(Prm-sb10)1Tcb* and NOD-*Tg(Prm-sb10)2Tcb*, but have been termed NOD-*PrmSB* L1 and L2 in the text. NOD-*SBtson* mice were mated to NOD-*PrmSB* mice and double-positive hemizygous male offspring (NOD-*SBtson^+^*/*PrmSB^+^*) were identified by PCR genotyping. NOD-*SBtson^+^/PrmSB^+^* males were backcrossed to wild-type NOD females to produce G1 mice, carrying potential transposon insertions. Mice carrying transposon insertions, which activated the polyA trap, were noninvasively identified by visualizing GFP expression in newborn mice under UV light with confirmation by the presence of GFP fluorescence in ear biopsies using a fluorescent stereomicroscope equipped with a UV filter. Experiments involving mice were approved by the St. Vincent’s Institute Animal Ethics Committee.

### Transposon copy number analysis

Southern blots were performed using standard protocols. An 898-bp probe specific for GFP labeled with α-^32^P-dCTP using the DECA prime II Random Priming DNA labeling kit (Ambion) was used to detect the presence of the *SB* transposon. Copy number was calculated by comparison with standards of known copy number.

### Transposon insertion site identification

Ligation-mediated PCR (LM-PCR) was performed based on previously described protocols (Largaespada and Collier 2008; Takeda *et al.* 2008; Horie *et al.* 2003; Devon *et al.* 1995). Briefly, 1 μg genomic DNA was digested with *Hae*III, *Alu*I, *Bfa*I, or *Nla*III (New England Biolabs), and splinkerettes (Table 1) compatible with appropriate blunt or cohesive ends were ligated to the digested genomic DNA fragments using T4 DNA ligase (New England Biolabs). The two oligonucleotides to produce the double-stranded splinkerette were annealed at a concentration of 50 μM in the presence of 100 mM NaCl by incubating at 95° for 5 min and then allowing the mixture to cool to room temperature in a heat block before ligation. A second digest, with *Xho*I or *Kpn*I, was used to remove ligated splinkerettes from transposon concatemer fragments. DNA was purified after each step using QiaQuick PCR Purification kit (Qiagen). Two rounds of PCR were performed using nested primers within the linker and transposon (Table 1) with the subsequent PCR product sequenced. Restriction enzymes, splinkerettes, and primers were used in the following six combinations (first digest; splinkerette; second digest; primer set 1; primer set 2): (i) and (ii) *Alu*I or *Hae*III; SplB-BLT; *Xho*I; T/JBA × Spl-P1; TJBI × Spl-P2; (iii) and (iv) *Alu*I or *Hae*III; SplB-BLT; *Kpn*I; TDR2 × Spl-P1; T/BAL × Spl-P2; (v) *Bfa*I; Bfa linker; *Kpn*I; LongIRDR(L2) × Linker primer; NewL1 × linker primer nested; and (vi) *Nla*III; Nla linker; *Xho*I; LongIRDR(R) × Linker primer; KJC1 × Linker primer nested. The resulting sequence was aligned to mouse genome build GRCm38 to identify the transposon-flanking genomic sequence (*i.e.*, insertion site) and determine which gene was disrupted based on current annotation for build GRCm38. The different combinations give a number of chances to identify genomic DNA adjacent to both the 3′ and 5′ ends of the transposon after transposition. Additional primers were designed for genotyping the transposon insertion site in established mutant mouse lines (Table 2).

**Table 1:** Oligonucleotides used as splinkerettes and primers for LM-PCR

Name*^a^*	Sequence	Purpose*^a^*
SplB-BLT	CGAATCGTAACCGTTCGTACGAGAATCGCTGTCCTCTCCAACGAGCCAAGG	Splinkerette
Spl-top	CCTTGGCTCGTTTTTTTTGCAAAAA	Splinkerette
Nla linker+	GTAATACGACTCACTATAGGGCTCCGCTTAAGGGACCATG	Splinkerette
Nla linker-	5′P-GTCCCTTAAGCGGAGCC-amino	Splinkerette
Bfa linker+	GTAATACGACTCACTATAGGGCTCCGCTTAAGGGAC	Splinkerette
Bfa linker-	5′P-TAGTCCCTTAAGCGGAG-amino	Splinkerette
Spl-P1	CGAATCGTAACCGTTCGTACGAGAA	LM-PCR
Spl-P2	TCGTACGAGAATCGCTGTCCTCTCC	Nested LM-PCR
T/JBA	TAACTGACCTTAAGACAGGGAATCTTTAC	LM-PCR (left)
TJB1	TTTACTCGGATTAAATGTCAGGAATG	Nested LM-PCR (left)
TDR2	CTGGAATTGTGATACAGTGAATTATAAGTG	LM-PCR (right)
T/BAL	CTTGTGTCATGCACAAAGTAGATGTCC	Nested LM-PCR (right)
LongIRDR(L2)	CTGGAATTTTCCAAGCTGTTTAAAGGCACAGTCAAC	LM-PCR (left)
NewL1	GACTTGTGTCATGCACAAAGTAGATGTCC	Nested LM-PCR (left)
LongIRDR(R)	GCTTGTGGAAGGCTACTCGAAATGTTTGACCC	LM-PCR (right)
KJC1	CCACTGGGAATGTGATGAAAGAAATAAAAGC	Nested LM-PCR (right)
Linker primer	GTAATACGACTCACTATAGGGC	LM-PCR
Linker primer nested	AGGGCTCCGCTTAAGGGAC	Nested LM-PCR

a Primer names and purpose are based on the previously described LM-PCR protocols (Keng *et al.* 2005; Largaespada and Collier 2008).

**Table 2:** Oligonucleotides used as primers for genotyping SB4 and SB7 mice

Line	Fwd	Rev	Size	Allele
SB4	AGCCCAGAAGACAACCCTCTTGT	AAAGGGGCGTGCGCTAAACA	123 bp	WT
SB4	CTTGTGTCATGCACAAAGTAGATGTCC	AAAGGGGCGTGCGCTAAACA	224 bp	SB
SB7	ATTCCACCAGTGATGTGCTGGTAAC	CCTTGAAATCATCCCGTGAGAGA	647 bp	WT
SB7	ATTCCACCAGTGATGTGCTGGTAAC	CTTGTGTCATGCACAAAGTAGATGTCC	632 bp	SB

### Gene expression analysis

RNA was extracted from tissues using Trizol (Invitrogen) and cDNA was synthesized using Superscript III (Invitrogen), both according to manufacturer’s instructions. For SB4 mice, RT-PCR was performed with oligos specific for *Slc16a10*: Exon 1 forward: 5′-GTGGTGCAACGGGTCGGTGT; Exon 2 reverse: 5′-GACACTCACGATGGGGCAGCA; Exon 6 reverse: 5′-ACAGAGCACAACACCCCCAACG; and *Actb* (forward: 5′-CGGTTCCGATGCCCTGAG; reverse: 5′-TGATCCACATCTGCTGGAAGG). For SB7 mice, RT-PCR was performed with primers in *GFP* (5′-CCCTGAGCAAAGACCCCAACGAGAAGC) and *Serinc1* exon 1 (reverse: 5′-TCGTCCTTTTTCTGGGCTTA) or exon 2 (reverse: 5′-ACTCCGACGAGCAAGAAAAG), followed by Sanger sequencing of products. Transposase expression was determined using quantitative real-time PCR using LightCycler Probe Master Reagent (Roche Diagnostics) and the following primer/UPL probe combination for SB transposase (*SB10*): Fwd: 5′-ACCACGCAGCCGTCATAC; Rev: 5′-CACCAAAGTACGTTCATCTCTAGG; UPL probe #12: GGAAGGAG; and *Hprt:* Fwd: 5′- TCCTCCTCAGACCGCTTTT; Rev: 5′- CCTGGTTCATCATCGCTAATC; UPL probe #95: AGTCCCAG. Data were normalized to *Hprt* expression. For SB7, *Serinc1* expression was determined by quantitative real-time PCR (LightCycler480, Roche) with the following primer/probe combination for *Serinc1*: (Fwd: 5′-GACGCGGCGGCGAT; Rev: 5′-GCATCGGCACAGCAAACAC; Taqman Probe: 5′-GCTGGATTCCGTGTTT) and normalization was performed using *Hprt* (Taqman assay: Mm01545399_m1). Data were normalized to *Hprt* expression.

### Diabetes monitoring

Mice were tested once per week for elevated urinary glucose using Diastix reagent strips (Bayer Diagnostics). Mice with a positive glycosuria reading (>110 mmol/L) and confirmed by a positive glucose reading (>15 mmol/L), using Advantage II Glucose Strips (Roche), were diagnosed as diabetic.

### Data availability

Mouse lines are available upon request.

## Results

### Generation of transposition events in NOD mice using *SB* transgenic NOD lines

To perform germline mutagenesis of the NOD mouse, we used two *SB* constructs previously used in mice (Figure 1A). The pRP1345 construct contains the transposase gene under the control of the proximal protamine 1 (*Prm1*) promoter, which restricts expression to spermatogenesis and limits transposon mutations to the germline, thus preventing somatic mutations (Fischer *et al.* 2001). The *SB* transposon construct (*sb-pTrans-SA-IRESLacZ-CAG-GFP-SD:Neo*) contains splice acceptor and donor sequence motifs encompassing a promoter trap comprising the *lacZ* gene, and a polyA trap with the gene encoding enhanced green fluorescent protein (*GFP*) driven by the CAG promoter (Horie *et al.* 2003). This construct enables efficient identification of mutant mice in which the transposon has inserted in a gene, as activation of the polyA trap results in ubiquitous GFP expression, *i.e.*, mutant mice fluoresce.

The transposase and transposon were introduced separately into NOD mice to establish independent transgenic NOD lines for each *SB* component. Briefly, *SB* transposon and *SB* transposase transgenic mice were produced by pronuclear injection of linearized constructs into fertilized NOD/Lt (NOD) oocytes. NOD-*Tg(Prm-sb10)* mice (called NOD-*PrmSB* hereafter) harbor the transposase; and NOD-*TgTn(sb-pTrans-SA-IRESLacZ-CAG-GFP-SD:Neo)* mice (called NOD-*SBtson* hereafter) harbor the transposon. The breeding scheme to generate mutant NOD mice is outlined in Figure 1B: mice from the two transgenic lines are mated, bringing together the two components of the *SB* system, and transposition occurs within the sperm cells of the double-positive hemizygous “seed” males. These NOD seed males are mated to wild-type NOD females to generate G_1_ litters. G_1_ pups with potential transposon-disrupted genes are efficiently identified before weaning by visualization of bodily GFP expression under UV light (Figure 1C). This early detection allows cage space to be minimized; only those litters that contain fluorescent pups are weaned and kept for additional analysis.

**Figure 2:**
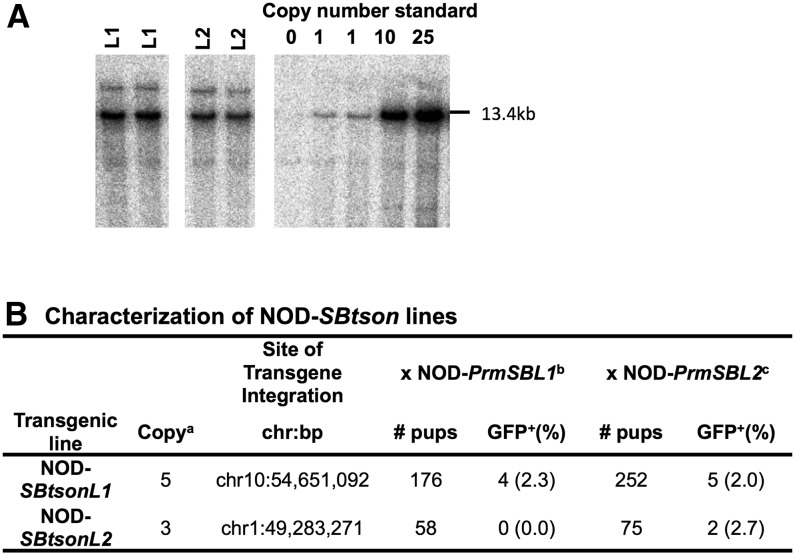
Characterization of NOD-*SBtson* lines and frequency of GFP-positive G_1_ pups. (A) Transgene copy number in NOD-*SBtson* lines was determined by Southern blot analysis using standard techniques and an 898-bp probe specific for *GFP* that detected a 13.4-kb band containing the transposon. Known amounts of transposon DNA were used to generate a standard curve for determining copy number. (B) Site of transgene integration was determined by LM-PCR (coordinates are based on genome build GRCm38). Number and percentage of GFP^+^ offspring for each breeding combination is shown. ^a^Number of copies of the transposon in the donor concatemer, determined by Southern blot as shown in (A). ^b^Offspring generated from seed males produced by breeding NOD-*SBtson* lines with NOD-*PrmSBL1*. ^c^Offspring generated from seed males produced by breeding NOD-*SBtson* line with NOD-*PrmSBL2*.

To assess the feasibility of the *SB* mutagenesis system in the NOD mouse strain, two NOD-*SBtson* lines were established for which the number of copies of the transposon within the transgene concatemer was determined by Southern Blot analysis (Figure 2A) and the site of transgene integration was determined by ligation-mediated PCR (LM-PCR) (Figure 2B). These lines were bred with two established NOD-*PrmSB* lines, in which expression of *SB* transposase in testes had been confirmed by quantitative RT-PCR (data not shown), to produce seed males. NOD seed males (*i.e.*, double-positive, hemizygous for both *SB* constructs) were backcrossed to NOD females to produce G_1_ mice. Three different breeding combinations of transposon/transposase transgenic strains gave rise to GFP-positive offspring at the rate of 2.0%, 2.3%, and 2.7% respectively (Figure 2B). A fourth combination did not produce any mutant pups (0%), but breeding of this fourth combination was stopped before reaching a similar total of G_1_ mice to the other combinations (Figure 2B). Eleven GFP-positive G_1_ mice were sired, confirming that these transgenic lines can facilitate *SB* transposition events on the NOD genetic background and resultant GFP-positive mice can be detected.

### Identification and prioritization of transposition sites in GFP-positive G_1_ NOD mice

LM-PCR followed by sequencing and genome alignment was used to identify the transposition sites for nine of the 11 GFP-positive mice (Table 3). Of the two GFP-positive mice that were not determined, one died before characterization. It was not clear why the second GFP-positive mouse was refractory to site identification by LM-PCR despite using six different restriction digest/PCR combinations. Consistent with the published >30% rate of local chromosomal hopping (Keng *et al.* 2005), seven of the nine identified transposition sites fell on the same chromosome as the transposon donor concatemer. Of the seven insertion sites derived from the NOD-*SBTsonL1* concatemer on chromosome (Chr) 10, five were on Chr10, with the other two identified on Chr1 and Chr4. The two insertion sites arising from the NOD-*SBTsonL2* concatemer on Chr1 were both mapped to Chr1. LM-PCR also indicated that each GFP-positive mouse contained a single, rather than multiple, transposon insertion site.

**Table 3:** *Sleeping Beauty* transposon insertion sites in GFP-positive G_1_ NOD mice

Mutant Mouse	NOD-*SBtson* Line	NOD-*PrmSB* Line	Site of Transposition Insertion (STI)*^a^*	Closest Gene in Correct Orientation	Gene Coordinates*^a^*	Position of STI with Respect to Gene
SB1	L1	L1	chr4:19,247,537	*Cnbd1*	chr4:18,860,454-19,122,526	125 kb 5′ of gene
SB2	L1	L1	chr10:30,040,682	*Rspo3*	chr10:29,453,107-29,535,867	505 kb 5′ of gene
SB3	L1	L1	chr10:54,845,255	*Msl3l2*	chr10:56,106,917-56,116,880	1.2 Mb 5′ of gene
SB4	L1	L1	chr10:40,122,645	*Slc16a10*	chr10:40,033,535-40,142,254	Intron 1
SB5*^c^*	L1	L2	n.d.	n.d.	n.d.	n.d.
SB6	L1	L2	n.d.	n.d.	n.d.	n.d.
SB7	L1	L2	chr10:57,537,126	*Serinc1*	chr10:57,515,775-57,532,529	4.6 kb 5′ of gene
SB8*^c^*	L1	L2	chr10:56,499,234	*AK018981*	chr10:56,504,501-56,505,287	5 kb 5′ of gene
SB9*^c^*	L1	L2	chr1:61,954,348	*Pard3bos1*	chr1:61,767,415-61,851,462	102 kb 5′ of gene*^b^*
SB10	L2	L2	chr1:49,094,718	*C230029F24Rik*	chr1:49,244,616-49,340,431	150 kb 5′
SB11	L2	L2	chr1:48,800,150	*Slc39a10*	chr1:46,807,544-46,853,509	1.9 Mb 5′ of gene

a Coordinates are based on genome build GRCm38.

b The SB9 transposon site of integration falls within intron 2 of *Pard3b* in the opposite orientation.

c SB5, SB8, and SB9 mice died of unknown causes before homozygous lines could be established.

n.d., not determined.

Once GFP-positive NOD mice and their transposon mutations are identified, there are two options. One option is to generate and monitor diabetes onset in cohorts of mice from every GFP-positive G_1_ mouse. Although such a full-scale phenotype-driven approach is aimed at identifying novel genes not suspected to play a role in diabetes, as well as known and putative candidate genes, this option requires substantial animal housing capacity and monitoring numerous cohorts for diabetes over a >200-d time course. Like many investigators, we have limited resources and this option was not feasible. However, the SB transposon mutagenesis strategy allows for a second option: prioritizing mutant mice based on the genes that are disrupted and establishing homozygous mutant lines for expression and diabetes monitoring. We therefore prioritized transposon-disrupted genes based on the following criteria:

1) The transposon insertion site is predicted to disrupt the gene in some way. Has the transposon inserted within an exon, an intron, or in a regulatory region? Is the mutation predicted to disrupt gene expression? An insertion that is predicted to completely abrogate expression of the normal gene product would be of high priority; however, in some instances a predicted hypomorphic allele may also be of value.
2) The gene is a known or putative candidate for a described mouse and/or human T1D susceptibility locus. T1D loci and candidate genes are curated in a searchable form at T1Dbase (*https://t1dbase.org/*) (Burren *et al.* 2011).
3) The gene, not previously considered as a putative T1D susceptibility gene, encodes a known protein involved in an immune-related molecular pathway that could affect T1D pathogenesis (*e.g.*, cytokine or chemokine signaling/regulation, pattern recognition pathways, costimulatory molecules, apoptosis, regulation of immune tolerance), but is not likely required for general immune cell development.
4) The gene, not previously considered as a putative T1D susceptibility gene, is expressed in relevant cells (*e.g.*, immune cell subsets, β cells) as determined in the first instance using gene expression databases [*e.g.*, Immunological Genome Project (https://www.immgen.org/)] (Heng *et al.* 2008) followed by additional expression analyses if needed.

Of the nine transposon insertion sites identified (Table 3), only four were located within or nearby (within 5 kb) annotated genes or expressed sequence tags (ESTs): SB4 in intron 1 of *Slc16a10*; SB7, 4.6 kb 5′ of *Serinc1*; SB8, 5 kb 5′ of *AK018981*; and SB9 in intron 2 of *Pard3b*. In particular, the transposon within *Pard3b* inserted in the opposite orientation to the direction of transcription of the gene, so it seems unlikely that it would disrupt expression of *Pard3b*, which encodes a cell polarity protein most highly expressed in the kidney (Kohjima *et al.* 2002). There is, however, an antisense gene, *Pard3bos1*, that overlaps *Pard3b* approximately 102 kb downstream of the transposon insertion site. Thus, it is more likely that the polyA gene-trap has been activated by splicing into this antisense gene. The remaining five transposons inserted in regions currently without any annotated genes, suggesting either the presence of unannotated genes or the presence of cryptic polyA sequence motifs as the GFP polyA trap was activated.

The following observations were made regarding the prioritization of the nine identified transposon insertion sites. Only one fell within a T1D susceptibility locus (*Pard3bos1* in *Idd5.1*) (Burren *et al.* 2011), but unfortunately this mouse (SB9) died before it could be bred. *AK018981* has no publicly available information except that it was sequenced from RNA isolated from adult mouse testes. Both *Serinc1* and *Slc16a10* have been previously disrupted in mice: *Serinc1* on a mixed C57BL/6 × 129 genetic background (Tang *et al.* 2010) and *Slc16a10* on a C57BL/6 background (Mariotta *et al.* 2012). Neither knockout mouse strain was reported to develop spontaneous disease. The strains either have not been analyzed for immunological phenotypes (*Slc16a10*) or have only been analyzed in small cohorts (n ≤ 4) with no differences observed (*Serinc1*). Despite this, *Serinc1* and *Slc16a10*, although not directly attributed in the literature with immune-related roles, have functions that could be postulated to affect immune cell responses and/or β-cell activity, which could be revealed in the context of the “sensitized” NOD genetic background (*i.e.*, the NOD mouse strain enables detection of mutated genes that increase or decrease diabetes incidence). Notably, both genes according to expression databases (The Immunological Genome Project and BioGPS) (Heng *et al.* 2008; Wu *et al.* 2009) were highly expressed in macrophages, an immune cell population with a key role in T1D pathogenesis (Driver *et al.* 2011; Jayasimhan *et al.* 2014). SB4 and SB7, which were both male G_1_ mice, did not carry the transposase construct; consequently, secondary jumping of the transposon was not possible in their offspring. SB4 and SB7 G_1_ mice were thus prioritized for establishment of new lines, bred to homozygosity, and investigated for the effect of their transposon insertions.

### Characterization of transposon effects in two prioritized GFP-positive G_1_ NOD mice

**Figure 3:**
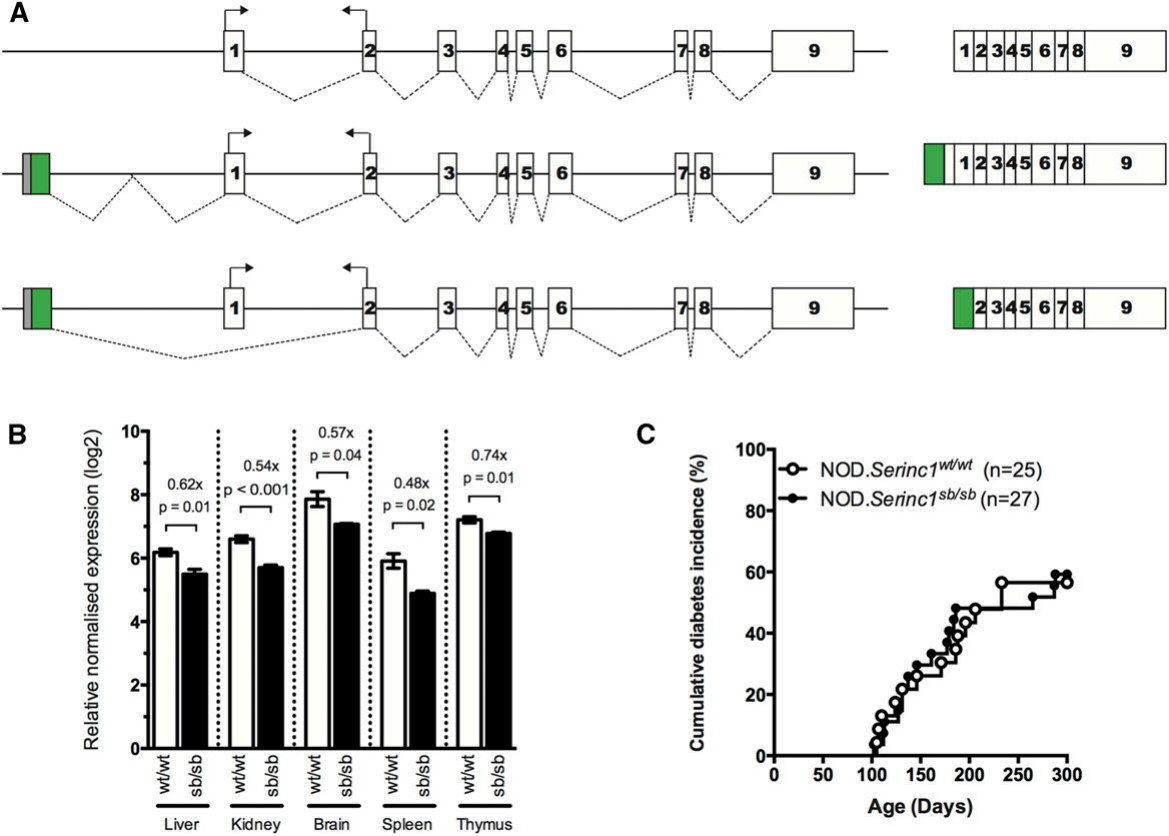
Analysis of *Serinc1* expression and diabetes incidence in SB7 mutant mice. (A) Schematic diagrams of *Serinc1* gene and transcripts with transposon insertion. *Serinc1* consists of nine exons and gives rise to a 2889-bp spliced transcript (top diagram). The transposon insertion occurs 4.6 kb 5′ of *Serinc1*. RT-PCR led to the identification of two fusion transcripts: one that splices from GFP (green) to sequence upstream of exon 1 resulting in otherwise normal splicing of *Serinc1* (middle diagram), and another that skips exon 1 (bottom diagram). (B) Expression analysis was performed using RNA isolated from tissues of wild-type (wt/wt) and homozygous mutant (sb/sb) littermates (n = 4). Expression was determined by quantitative real-time PCR. Exon locations of *Serinc1* primers are indicated by arrows in (A). Error bars represent ± SEM. Statistical significance for the difference in expression was obtained using pairwise *t*-tests. (C) The cumulative diabetes incidence was determined for age-matched female cohorts monitored concurrently. Pairwise comparisons of diabetes incidence curves were performed using the log-rank test.

The transposon insertion site in the SB7 mouse (NOD.*Serinc1^Tn(sb-Trans-SA-IRESLacZ-CAG-GFP-SD:Neo)1.7Tcb^*) localized 4.6 kb upstream of *Serinc1* (Table 3, Figure 3A). SERINC1 facilitates the synthesis of serine-derived lipids, including the essential membrane lipids phosphatidylserine and sphingolipid (Inuzuka *et al.* 2005). These are important components of membrane structures known as “ordered membrane domains” or “lipid rafts,” which are required for appropriate signaling in immune cells (Szoor *et al.* 2010; Yabas *et al.* 2011). Due to the position of the transposon insertion, we postulated that, rather than completely disrupting expression of *Serinc1*, the mutation may affect gene regulation. RT-PCR analysis of homozygous SB7 splenic RNA using primers within *GFP* and *Serinc1* identified two fusion transcripts in addition to the normal transcript. The first contains *GFP* spliced to sequence upstream of exon 1 with normal splicing of the entire gene. The second contains *GFP* spliced directly to exon 2 of *Serinc1* (Figure 3A). The skipped exon 1 encodes the first 13 amino acids of the *Serinc1* coding sequence. The production of any SERINC1 protein from these fusion transcripts is unlikely because there is a stop codon following the *GFP* sequence and no obvious internal ribosomal entry site prior to the *Serinc1* sequence, but this still remains to be tested. In either case, the presence of the transposon insertion results in a relatively small, but significant, decrease in expression of *Serinc1* as measured by quantitative RT-PCR using a Taqman probe spanning the exon 1/2 splice junction (Figure 3B). This splice junction is used in both the “normal” *Serinc1* and the fusion transcript that includes exon 1; therefore, the reduction in “normal” *Serinc1* expression is greater than measured by this assay. These analyses suggest that, rather than completely disrupting expression of *Serinc1*, this mutation modulates expression and represents a hypomorphic allele. Nonetheless, homozygous mutant SB7 mice exhibited a similar diabetes incidence compared to wild-type littermate females (Figure 3C), indicating that a minor reduction in *Serinc1* expression does not affect T1D pathogenesis.

**Figure 4:**
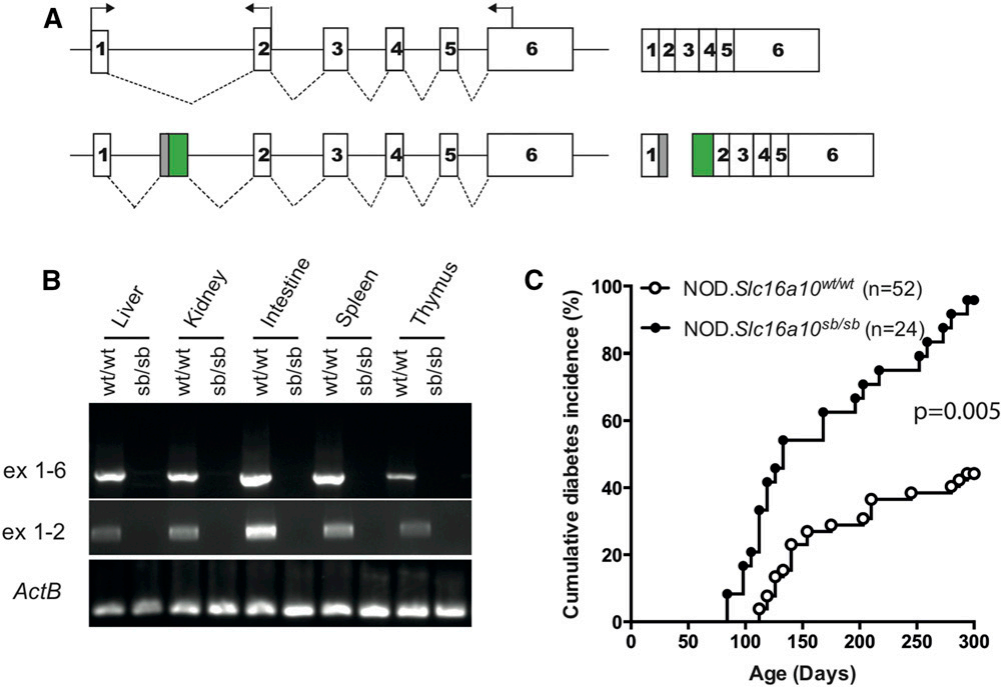
Analysis of *Slc16a10* expression and diabetes incidence in SB4 mutant mice. (A) Schematic diagrams of *Slc16a10* gene and transcript with transposon insertion. *Slc16a10* consists of six exons and gives rise to a 5394-bp spliced transcript (top diagram). The transposon insertion occurs within intron 1 and is predicted to prevent splicing between exons 1 and 2, instead generating a truncated transcript [exon 1 - transposon (gray box)] and a transcript initiated by the CAG promoter and GFP gene [transposon (green box) - exon 2] (bottom diagram). (B) Detection of transcript expression was performed by RT-PCR using RNA isolated from tissues of wild-type (wt/wt) and homozygous mutant (sb/sb) littermates. Spliced products for exon 1/exon 2 or exon 1/exon 6 were not detected in any tissues tested from homozygous mutant mice. Expression analysis is representative of three mice per genotype. (C) The cumulative diabetes incidence was determined for age-matched female cohorts monitored concurrently. Pairwise comparisons of diabetes incidence curves were performed using the log-rank test.

The transposon insertion identified in the SB4 mouse (NOD.*Slc16a10^Tn(sb-Trans-SA-IRESLacZ-CAG-GFP-SD:Neo)1.4Tcb^*) localizes to intron 1 of *Slc16a10* (Table 3, Figure 4A), which encodes the aromatic amino acid transporter SLC16A10 (also known as TAT1) (Mariotta *et al.* 2012; Ramadan *et al.* 2006). It is becoming increasingly evident that regulation of amino acid transport is crucial for the proper regulation of immune cell activation and function (Nakaya *et al.* 2014; Sinclair *et al.* 2013; Thompson *et al.* 2008), and also impacts glycemic control (Jiang *et al.* 2015). We predicted that the strong splice acceptor encoded by the transposon would result in splicing from exon 1 of *Slc16a10* into the transposon sequence, thus truncating the *Slc16a10* transcript. Expression analysis of homozygous mutant SB4 mice showed that the *Slc16a10* transcript was not detected (Figure 4B), which was associated with a significant increase in diabetes incidence compared to wild-type littermate females (Figure 4C). This result demonstrates that SB transposon mutagenesis can be used to identify novel genes that affect T1D pathogenesis. Moreover, SB4 is a promising mutant mouse line for investigating the role of *Slc16a10* and aromatic amino acid transport in macrophage function and the development of T1D in NOD mice.

## Discussion

We demonstrate here that *SB* transposon mutagenesis can successfully generate both null and hypomorphic mutations in the NOD mouse. A significant advantage of this strategy is the use of the GFP reporter to screen out, before weaning, those mice unlikely to be carrying a functional mutation, thereby significantly reducing mouse handling and housing. Mutation sites can then be determined in individual GFP-positive mice and prioritized for further analysis based on the requirements of the investigator. We used our prioritization criteria to select two mutant NOD mice (SB4 and SB7) for further characterization and subsequently found that disruption of *Slc16a10* expression in NOD mice resulted in an increased T1D incidence. Although further studies are required to determine how *Slc16a10* and amino acid transport contributes to diabetes pathogenesis, this result indicates that SB transposon mutagenesis can be used as a complementary approach to other T1D gene discovery strategies.

We observed 2–3% fluorescent G_1_ offspring, which is lower than that reported for a similar mutagenesis scheme using the same transposon construct (∼7%) (Horie *et al.* 2003). If one aims for a single transposition event per G_1_ offspring, it would be expected that ∼20% of the G_1_ offspring should fluoresce [*i.e.*, ∼40% of the mouse genome contains exons/introns (Sakharkar *et al.* 2005), with a 50% chance of the transposon landing in the correct orientation to trap the polyA sequence motif]. There are a number of possibilities that could explain a lower rate of fluorescent G_1_ offspring, pointing to improvements that can be made to our screen. The NOD-*SBtson* “mutator” transgenic lines we generated harbored few copies of the transposon. A higher copy number in the donor transposon concatemer may increase mutation efficiency due to more “jumping” transposons in the sperm of NOD seed males (Geurts *et al.* 2006). Use of a more efficient transposase could also increase transposition efficiency. As the first described transposon for use in vertebrates, the *SB* system has been the most widely used and developed. Improvements from the first-generation *SB* transposases, such as that used in our study, have seen 100-fold increases in transposition efficiency (Mates *et al.* 2009).

Trying to obtain too high of a mutation rate, however, is not necessarily favorable. For example, increasing transposon copy number by using transgenic mice with large transposon concatemers (>30 copies) may lead to local chromosomal rearrangements in subsequent offspring and complicate characterization of causative transposon mutations (Geurts *et al.* 2006). Increasing transposon number and/or transposase efficiency will also lead to GFP-positive offspring with multiple gene mutations. It would then require additional work to identify and confirm all the gene mutations in a given mouse, as well as additional breeding to segregate mutations and test mutant mice with only one gene mutation, all of which increases breeding times and cage costs. Thus, an advantage of a lower efficiency is that it leads to mice with only one gene mutation, eliminating the need for extensive segregation analysis and facilitating more efficient gene identification and prioritization. Although we did not establish and characterize all of our mutant mouse lines due to limited resources, cryopreservation of sperm from mutant male mice could be used to allow archiving of mutants for future investigation. Empirically, it will be up to an individual laboratory to determine how many offspring they can efficiently screen and how they will prioritize mutant mice for subsequent analysis based on the mutation rate of their transgenic lines and available resources.

Interestingly, several of our transposon insertion sites mapped to regions at some distance from annotated genes. Although it is possible that the GFP could be activated by splicing into a cryptic polyA site, polyA gene trap strategies have been successfully used to identify novel unannotated genes (Zambrowicz *et al.* 1998). However, it may be difficult to predict *a priori* if an unannotated gene will be of interest, especially if it has little to no sequence homology with known genes. In this regard, the *SB* mutagenesis approach enables a phenotype-driven (*i.e.*, forward genetics) approach to test novel genes that might not otherwise be targeted using a candidate gene approach (*i.e.*, reverse genetics). *SB* mutagenesis may also benefit characterization of regions (*e.g.*, *Idd* loci) that are known to contain putative susceptibility genes, but for which the “natural” causative alleles have not yet been identified. Saturation mutagenesis could effectively be performed in these regions by generating a transgenic NOD mouse line containing a transposon concatemer near the region of interest and taking advantage of the propensity of *SB* transposons to reintegrate close to the donor transposon concatemer (Keng *et al.* 2005). Nonetheless, investigating unannotated genes is riskier (*i.e.*, the disrupted gene may not affect T1D) or more costly in the case of saturation mutagenesis (*i.e.*, more mutant lines need to be generated and characterized). Hence, prioritizing genes based on function postulated to contribute to T1D pathogenesis may be more favorable to most labs.

Transposon mutagenesis is one of a range of techniques that can be used to identify gene variants that affect development of T1D. Conventionally, “artificial” null alleles for candidate genes have been generated in other strains and bred onto the NOD background by serial backcrossing. This approach, however, results in the null allele being encompassed by a “hitchhiking” congenic interval from the other strain, which may also affect T1D susceptibility (Simpfendorfer *et al.* 2015; Armstrong *et al.* 2006; Leiter 2002; Kanagawa *et al.* 2000). Although NOD ES cell lines are available (Hanna *et al.* 2009; Nichols *et al.* 2009; Ohta *et al.* 2009), there are relatively few reports of their use in targeting genes (Kamanaka *et al.* 2009; Morgan *et al.* 2013). Alternatively, random mutagenesis using *N*-ethyl-*N*-nitrosourea (ENU) does not require ES cells or prior knowledge about candidate genes. ENU mutagenesis, however, creates tens to hundreds of mutations per mouse. Substantial breeding and sequencing, more than for SB transposon mutagenesis, would be required to segregate and test individual ENU mutations for their effect on T1D susceptibility (Hoyne and Goodnow 2006). Finally, emerging gene-editing techniques using the CRISPR-Cas9 system (Ran *et al.* 2013) can facilitate hypothesis-driven investigation of known and putative candidate genes in NOD mice (Ran *et al.* 2013; Li *et al.* 2014). Modifications of the CRISPR-Cas9 system also allow a range of strategies to be used, including the production of conditional alleles, insertion of reporters, activation or repression of alleles, and specific gene editing allowing the recreation of particular variants affecting immune function (Pelletier *et al.* 2015). Nonetheless, this approach requires genes to be specifically targeted, whereas SB transposon mutagenesis is random and may identify genes not otherwise considered. Thus, a combination of different approaches for gene discovery and characterization of allelic effects is available and will likely prove useful for understanding the genetic architecture of T1D.

The increasing number of causative “natural” alleles identified in human populations and inbred mouse strains will undoubtedly aid our understanding and prediction of genetic risk for T1D, as well as aid future clinical trials in selecting appropriate patient treatment groups based on their genetic profile (Bluestone *et al.* 2010; Polychronakos and Li 2011). Nonetheless, many of the identified T1D loci and underlying causative alleles have subtle biological effects that are not therapeutically amenable or are difficult to investigate due to tissue availability. Generating random “artificial” null alleles in the NOD mouse provides an alternative strategy to test and identify both putative and novel genes that: (i) have larger diabetes effects when more grossly disrupted; (ii) represent potential drug targets; and (iii) are less likely to be identified in population-based studies of natural variation. The NOD mouse exhibits a number of immunological abnormalities that are associated with T1D pathogenesis and provides a “sensitized” background to investigate the effect of artificial mutations upon the development of T1D (Driver *et al.* 2011; Ridgway *et al.* 2008; Jayasimhan *et al.* 2014). Our study indicates that *SB* transposon mutagenesis in NOD mice is feasible and provides a new strategy that combines the advantage of both forward genetics (random mutagenesis) and reverse genetics (gene prioritization) for the potential discovery of new genes that affect T1D pathogenesis.

## References

[bib1] Araki M. Chung D. Liu S. Rainbow D. B. Chamberlain G., 2009 Genetic evidence that the differential expression of the ligand-independent isoform of CTLA-4 is the molecular basis of the *Idd5.1* type 1 diabetes region in nonobese diabetic mice. J. Immunol. 183: 5146–5157. 19783679 10.4049/jimmunol.0802610 PMC2871291

[bib2] Armstrong N. J. Brodnicki T. C. Speed T. P., 2006 Mind the gap: analysis of marker-assisted breeding strategies for inbred mouse strains. Mamm. Genome 17: 273–287. 16596449 10.1007/s00335-005-0123-y

[bib3] Bluestone J. A. Herold K. Eisenbarth G., 2010 Genetics, pathogenesis and clinical interventions in type 1 diabetes. Nature 464: 1293–1300. 20432533 10.1038/nature08933 PMC4959889

[bib4] Brodnicki T. C. Quirk F. Morahan G., 2003 A susceptibility allele from a non-diabetes-prone mouse strain accelerates diabetes in NOD congenic mice. Diabetes 52: 218–222. 12502517 10.2337/diabetes.52.1.218

[bib5] Burren O. S. Adlem E. C. Achuthan P. Christensen M. Coulson R. M., 2011 T1DBase: update 2011, organization and presentation of large-scale data sets for type 1 diabetes research. Nucleic Acids Res. 39(Database issue): D997–D1001. 20937630 10.1093/nar/gkq912 PMC3013780

[bib6] Carlson C. M. Dupuy A. J. Fritz S. Roberg-Perez K. J. Fletcher C. F., 2003 Transposon mutagenesis of the mouse germline. Genetics 165: 243–256. 14504232 10.1093/genetics/165.1.243 PMC1462753

[bib7] Devon R. S. Porteous D. J. Brookes A. J., 1995 Splinkerettes–improved vectorettes for greater efficiency in PCR walking. Nucleic Acids Res. 23: 1644–1645. 7784225 10.1093/nar/23.9.1644 PMC306912

[bib8] Driver J. P. Serreze D. V. Chen Y. G., 2011 Mouse models for the study of autoimmune type 1 diabetes: a NOD to similarities and differences to human disease. Semin. Immunopathol. 33: 67–87. 20424843 10.1007/s00281-010-0204-1

[bib9] Dupuy A. J. Rogers L. M. Kim J. Nannapaneni K. Starr T. K., 2009 A modified *Sleeping Beauty* transposon system that can be used to model a wide variety of human cancers in mice. Cancer Res. 69: 8150–8156. 19808965 10.1158/0008-5472.CAN-09-1135 PMC3700628

[bib10] Ermann J. Glimcher L. H., 2012 After GWAS: mice to the rescue? Curr. Opin. Immunol. 24: 564–570. 23031443 10.1016/j.coi.2012.09.005 PMC3631559

[bib11] Fischer S. E. Wienholds E. Plasterk R. H., 2001 Regulated transposition of a fish transposon in the mouse germ line. Proc. Natl. Acad. Sci. USA 98: 6759–6764. 11381141 10.1073/pnas.121569298 PMC34426

[bib12] Geurts A. M. Collier L. S. Geurts J. L. Oseth L. L. Bell M. L., 2006 Gene mutations and genomic rearrangements in the mouse as a result of transposon mobilization from chromosomal concatemers. PLoS Genet. 2: e156. 17009875 10.1371/journal.pgen.0020156 PMC1584263

[bib13] Ghosh S. Palmer S. M. Rodrigues N. R. Cordell H. J. Hearne C. M., 1993 Polygenic control of autoimmune diabetes in nonobese diabetic mice. Nat. Genet. 4: 404–409. 8401590 10.1038/ng0893-404

[bib14] Hamilton-Williams E. E. Serreze D. V. Charlton B. Johnson E. A. Marron M. P., 2001 Transgenic rescue implicates β2-microglobulin as a diabetes susceptibility gene in nonobese diabetic (NOD) mice. Proc. Natl. Acad. Sci. USA 98: 11533–11538. 11572996 10.1073/pnas.191383798 PMC58764

[bib15] Hanna J. Markoulaki S. Mitalipova M. Cheng A. W. Cassady J. P., 2009 Metastable pluripotent states in NOD-mouse-derived ESCs. Cell Stem Cell 4: 513–524. 19427283 10.1016/j.stem.2009.04.015 PMC2714944

[bib16] Heng T.S. Painter M.W., and Immunological Genome Project Consortium, 2008 The Immunological Genome Project: networks of gene expression in immune cells. Nat. Immunol. 9: 1091–1094. 18800157 10.1038/ni1008-1091

[bib17] Horie K. Yusa K. Yae K. Odajima J. Fischer S. E., 2003 Characterization of *Sleeping Beauty* transposition and its application to genetic screening in mice. Mol. Cell. Biol. 23: 9189–9207. 14645530 10.1128/MCB.23.24.9189-9207.2003 PMC309709

[bib18] Hoyne G. F. Goodnow C. C., 2006 The use of genomewide ENU mutagenesis screens to unravel complex mammalian traits: identifying genes that regulate organ-specific and systemic autoimmunity. Immunol. Rev. 210: 27–39. 16623762 10.1111/j.0105-2896.2006.00363.x

[bib19] Hung M. S. Avner P. Rogner U. C., 2006 Identification of the transcription factor *Arntl2* as a candidate gene for the type 1 diabetes locus *Idd6*. Hum. Mol. Genet. 15: 2732–2742. 16893914 10.1093/hmg/ddl209

[bib20] Inuzuka M. Hayakawa M. Ingi T., 2005 Serinc, an activity-regulated protein family, incorporates serine into membrane lipid synthesis. J. Biol. Chem. 280: 35776–35783. 16120614 10.1074/jbc.M505712200

[bib21] Ivics Z. Hackett P. B. Plasterk R. H. Izsvak Z., 1997 Molecular reconstruction of *Sleeping Beauty*, a T*c1*-like transposon from fish, and its transposition in human cells. Cell 91: 501–510. 9390559 10.1016/s0092-8674(00)80436-5

[bib22] Izsvak Z. Ivics Z., 2004 *Sleeping Beauty* transposition: biology and applications for molecular therapy. Mol. Ther. 9: 147–156. 14759798 10.1016/j.ymthe.2003.11.009

[bib23] Jayasimhan A. Mansour K. P. Slattery R. M., 2014 Advances in our understanding of the pathophysiology of type 1 diabetes: lessons from the NOD mouse. Clin. Sci. (Lond.) 126: 1–18. 24020444 10.1042/CS20120627

[bib24] Jiang Y. Rose A. J. Sijmonsma T. P. Broer A. Pfenninger A., 2015 Mice lacking neutral amino acid transporter B^0^AT1 (*Slc6a19*) have elevated levels of FGF21 and GLP-1 and improved glycaemic control. Mol. Metab. 4: 406–417. 25973388 10.1016/j.molmet.2015.02.003 PMC4421019

[bib25] Kamanaka M. Rainbow D. Schuster-Gossler K. Eynon E. E. Chervonsky A. V., 2009 Amino acid polymorphisms altering the glycosylation of IL-2 do not protect from type 1 diabetes in the NOD mouse. Proc. Natl. Acad. Sci. USA 106: 11236–11240. 19549859 10.1073/pnas.0904780106 PMC2700154

[bib26] Kanagawa O. Xu G. Tevaarwerk A. Vaupel B. A., 2000 Protection of nonobese diabetic mice from diabetes by gene(s) closely linked to IFN-γ receptor loci. J. Immunol. 164: 3919–3923. 10725755 10.4049/jimmunol.164.7.3919

[bib27] Keng V. W. Yae K. Hayakawa T. Mizuno S. Uno Y., 2005 Region-specific saturation germline mutagenesis in mice using the *Sleeping Beauty* transposon system. Nat. Methods 2: 763–769. 16179923 10.1038/nmeth795

[bib28] Kissler S. Stern P. Takahashi K. Hunter K. Peterson L. B., 2006 *In vivo* RNA interference demonstrates a role for Nramp1 in modifying susceptibility to type 1 diabetes. Nat. Genet. 38: 479–483. 16550170 10.1038/ng1766

[bib29] Kohjima M. Noda Y. Takeya R. Saito N. Takeuchi K., 2002 PAR3beta, a novel homologue of the cell polarity protein PAR3, localizes to tight junctions. Biochem. Biophys. Res. Commun. 299: 641–646. 12459187 10.1016/s0006-291x(02)02698-0

[bib30] Laloraya M. Davoodi-Semiromi A. Kumar G. P. McDuffie M. She J. X., 2006 Impaired Crkl expression contributes to the defective DNA binding of *Stat5b* in nonobese diabetic mice. Diabetes 55: 734–741. 16505237 10.2337/diabetes.55.03.06.db05-1059

[bib31] Largaespada D. A. Collier L. S., 2008 Transposon-mediated mutagenesis in somatic cells: identification of transposon-genomic DNA junctions. Methods Mol. Biol. 435: 95–108. 18370070 10.1007/978-1-59745-232-8_7 PMC3517914

[bib32] Leiter E. H., 2002 Mice with targeted gene disruptions or gene insertions for diabetes research: problems, pitfalls, and potential solutions. Diabetologia 45: 296–308. 11914735 10.1007/s00125-001-0743-z

[bib33] Li F. Cowley D. O. Banner D. Holle E. Zhang L., 2014 Efficient genetic manipulation of the *NOD-Rag12/2IL2RgammaC-null* mouse by combining in vitro fertilization and CRISPR/Cas9 technology. Sci. Rep. 4: 5290. 24936832 10.1038/srep05290 PMC4894429

[bib34] Mariotta L. Ramadan T. Singer D. Guetg A. Herzog B., 2012 T-type amino acid transporter TAT1 (*Slc16a10*) is essential for extracellular aromatic amino acid homeostasis control. J. Physiol. 590(Pt 24): 6413–6424. 23045339 10.1113/jphysiol.2012.239574 PMC3533202

[bib35] Marshall V. M. Allison J. Templeton T. Foote S. J., 2004 Generation of BAC transgenic mice. Methods Mol. Biol. 256: 159–182. 15024166 10.1385/1-59259-753-X:159

[bib36] Mates L. Chuah M. K. Belay E. Jerchow B. Manoj N., 2009 Molecular evolution of a novel hyperactive *Sleeping Beauty* transposase enables robust stable gene transfer in vertebrates. Nat. Genet. 41: 753–761. 19412179 10.1038/ng.343

[bib37] McAleer M. A. Reifsnyder P. Palmer S. M. Prochazka M. Love J. M., 1995 Crosses of NOD mice with the related NON strain. A polygenic model for IDDM. Diabetes 44: 1186–1195. 7556956 10.2337/diab.44.10.1186

[bib38] McGuire H. M. Vogelzang A. Hill N. Flodstrom-Tullberg M. Sprent J., 2009 Loss of parity between IL-2 and IL-21 in the NOD *Idd3* locus. Proc. Natl. Acad. Sci. USA 106: 19438–19443. 19880748 10.1073/pnas.0903561106 PMC2780811

[bib39] Morgan M. A. Muller P. S. Mould A. Newland S. A. Nichols J., 2013 The nonconventional MHC class II molecule DM governs diabetes susceptibility in NOD mice. PLoS One 8: e56738. 23418596 10.1371/journal.pone.0056738 PMC3572069

[bib40] Moriarity B. S. Largaespada D. A., 2015 *Sleeping Beauty* transposon insertional mutagenesis based mouse models for cancer gene discovery. Curr. Opin. Genet. Dev. 30: 66–72. 26051241 10.1016/j.gde.2015.04.007 PMC4900178

[bib41] Nakaya M. Xiao Y. Zhou X. Chang J. H. Chang M., 2014 Inflammatory T cell responses rely on amino acid transporter ASCT2 facilitation of glutamine uptake and mTORC1 kinase activation. Immunity 40: 692–705. 24792914 10.1016/j.immuni.2014.04.007 PMC4074507

[bib42] Nichols J. Jones K. Phillips J. M. Newland S. A. Roode M., 2009 Validated germline-competent embryonic stem cell lines from nonobese diabetic mice. Nat. Med. 15: 814–818. 19491843 10.1038/nm.1996

[bib43] Ohta H. Ohinata Y. Ikawa M. Morioka Y. Sakaide Y., 2009 Male germline and embryonic stem cell lines from NOD mice: efficient derivation of GS cells from a nonpermissive strain for ES cell derivation. Biol. Reprod. 81: 1147–1153. 19726737 10.1095/biolreprod.109.079368

[bib44] Pelletier S. Gingras S. Green D. R., 2015 Mouse genome engineering via CRISPR-Cas9 for study of immune function. Immunity 42: 18–27. 25607456 10.1016/j.immuni.2015.01.004 PMC4720985

[bib45] Pociot F. Akolkar B. Concannon P. Erlich H. A. Julier C., 2010 Genetics of type 1 diabetes: what’s next? Diabetes 59: 1561–1571. 20587799 10.2337/db10-0076 PMC2889752

[bib46] Polychronakos C. Li Q., 2011 Understanding type 1 diabetes through genetics: advances and prospects. Nat. Rev. Genet. 12: 781–792. 22005987 10.1038/nrg3069

[bib47] Ramadan T. Camargo S. M. Summa V. Hunziker P. Chesnov S., 2006 Basolateral aromatic amino acid transporter TAT1 (*Slc16a10*) functions as an efflux pathway. J. Cell. Physiol. 206: 771–779. 16245314 10.1002/jcp.20531

[bib48] Ran F. A. Hsu P. D. Wright J. Agarwala V. Scott D. A., 2013 Genome engineering using the CRISPR-Cas9 system. Nat. Protoc. 8: 2281–2308. 24157548 10.1038/nprot.2013.143 PMC3969860

[bib49] Razavi R. Chan Y. Afifiyan F. N. Liu X. J. Wan X., 2006 TRPV1+ sensory neurons control β cell stress and islet inflammation in autoimmune disease. Cell 127: 1123–1135. 17174891 10.1016/j.cell.2006.10.038

[bib50] Ridgway W. M. Peterson L. B. Todd J. A. Rainbow D. B. Healy B., 2008 Gene-gene interactions in the NOD mouse model of type 1 diabetes. Adv. Immunol. 100: 151–175. 19111166 10.1016/S0065-2776(08)00806-7

[bib51] Sakharkar M. K. Perumal B. S. Sakharkar K. R. Kangueane P., 2005 An analysis on gene architecture in human and mouse genomes. In Silico Biol. 5: 347–365. 16268780

[bib52] Simpfendorfer K. R. Strugnell R. A. Brodnicki T. C. Wijburg O. L., 2015 Increased autoimmune diabetes in *pIgR*-deficient NOD mice is due to a “Hitchhiking” interval that refines the genetic effect of *Idd5.4*. PLoS One 10: e0121979. 25835383 10.1371/journal.pone.0121979 PMC4383422

[bib53] Sinclair L. V. Rolf J. Emslie E. Shi Y. B. Taylor P. M., 2013 Control of amino-acid transport by antigen receptors coordinates the metabolic reprogramming essential for T cell differentiation. Nat. Immunol. 14: 500–508. 23525088 10.1038/ni.2556 PMC3672957

[bib54] Szoor A. Szollosi J. Vereb G., 2010 Rafts and the battleships of defense: the multifaceted microdomains for positive and negative signals in immune cells. Immunol. Lett. 130: 2–12. 20026358 10.1016/j.imlet.2009.12.016

[bib55] Takeda J. Izsvak Z. Ivics Z., 2008 Insertional mutagenesis of the mouse germline with *Sleeping Beauty* transposition. Methods Mol. Biol. 435: 109–125. 18370071 10.1007/978-1-59745-232-8_8

[bib56] Tan I. K. Mackin L. Wang N. Papenfuss A. T. Elso C. M., 2010 A recombination hotspot leads to sequence variability within a novel gene (*AK005651*) and contributes to type 1 diabetes susceptibility. Genome Res. 20: 1629–1638. 21051460 10.1101/gr.101881.109 PMC2989989

[bib57] Tang T. Li L. Tang J. Li Y. Lin W. Y., 2010 A mouse knockout library for secreted and transmembrane proteins. Nat. Biotechnol. 28: 749–755. 20562862 10.1038/nbt.1644

[bib58] Thompson R. W. Pesce J. T. Ramalingam T. Wilson M. S. White S., 2008 Cationic amino acid transporter-2 regulates immunity by modulating arginase activity. PLoS Pathog. 4: e1000023. 18369473 10.1371/journal.ppat.1000023 PMC2265428

[bib59] Wang N. Elso C. M. Mackin L. Mannering S. I. Strugnell R. A., 2014 Congenic mice reveal genetic epistasis and overlapping disease loci for autoimmune diabetes and listeriosis. Immunogenetics 66: 501–506. 24906421 10.1007/s00251-014-0782-5

[bib60] Wu C. Orozco C. Boyer J. Leglise M. Goodale J., 2009 BioGPS: an extensible and customizable portal for querying and organizing gene annotation resources. Genome Biol. 10: R130. 19919682 10.1186/gb-2009-10-11-r130 PMC3091323

[bib61] Yabas M. Teh C. E. Frankenreiter S. Lal D. Roots C. M., 2011 ATP11C is critical for the internalization of phosphatidylserine and differentiation of B lymphocytes. Nat. Immunol. 12: 441–449. 21423173 10.1038/ni.2011 PMC3272780

[bib62] Yamanouchi J. Rainbow D. Serra P. Howlett S. Hunter K., 2007 Interleukin-2 gene variation impairs regulatory T cell function and causes autoimmunity. Nat. Genet. 39: 329–337. 17277778 10.1038/ng1958 PMC2886969

[bib63] Zambrowicz B. P. Friedrich G. A. Buxton E. C. Lilleberg S. L. Person C., 1998 Disruption and sequence identification of 2,000 genes in mouse embryonic stem cells. Nature 392: 608–611. 9560157 10.1038/33423

